# Supplementary material for: Absence of TGFβ signaling in retinal microglia induces retinal degeneration and exacerbates choroidal neovascularization
Source: eLife. 2019 Jan 22;8:e42049. doi: 10.7554/eLife.42049 (PMC6342522; doi:10.7554/eLife.42049)
Supplement: Supplementary file 1. [file elife-42049-supp1.docx]

**Supplementary Table 1. Sequences of oligonucleotide primers used in polymerase chain reaction (PCR) assays**

| Name | Reverse (5’-3’) | Forward (5’-3’) |
| --- | --- | --- |
| Rps13 | CCTCCCCGAGGATCTCTAC | GAGCAGAGGCTGTGGATGAC |
| Ctss | CTGGGTAAGAGCAATAACTAGC | GGCAATGTGAATCATGGTGTTC |
| Il1b | GCTGGAGAGTGTGGATCCC | GACACGGATTCCATGGTGAAG |
| Trem2 | CACGTACCTCCGGGTCCAG | CCACCTCCATTCTTCTCCTC |
| Bdnf | CAGTGTACATACACAGGAAGTG | GCAGTATTTCTACGAGACCAAG |
| Cd11b | GCAGGAGTCGTATGTGAGG | TTACTGAGGTGGGGCGTCT |
| Cx3cr1 | GTTGCCTCAACCCCTTTATCT | CAGGAGAGACCCATCTCCC |
| P2ry12 | GTTCTACGTGAAGGAGAGCA | CTACATTGGGGTCTCTTCGC |
| P2ry13 | CTGTAGGTTAGAAAACCAGCTG | CAGGCTAGGGTGATGTTGTC |
| Apoe | GATTCTCCAGGGCCACTGG | GGTGCGTGAGCACATGGAG |
| Tmem119 | GGTCACTGAAGAGAAGCTGG | CTCTGTTGCAGGCACAGGG |
| Siglech | CAGCCCATGTCTCTGGAAG | CTAATCCATGACAGCGTGTG |
| Cd68 | CACCTTGACCTGCTCTCTC | TCAGAGGGGCTGGTAGGTT |
| Cd74 | CACAGGGTGACTTGACCCAG | CCCAAGTGCGACGAGAACG |
| Ccl2 | CCTGGATCGGAACCAAATGA | CTAGTTCACTGTCACACTGG |
| Ccl8 | CAAGGCTGCAGAATTTGAGAC | CACCTGCTGCTTTCATGTAC |
| Igf1 | CTACTTGTGTTCTTCAAATGTAC | CTGAGGAGACTGGAGATGTAC |
| H2-Aa | GCCGGTTCTGAAACACTGGG | CATAAAGGCCCTGGGTGTCTG |
| Gfap | CCAGGATCTACTCAACGTTAAG | GCATCTCCACAGTCTTTACCAC |
| Pdgfa | CCTCACATCTGTCTCCTCCT | GCCAGCCTTCACGGGTCC |
| Tgfbr2-E3 | CTCTGGAGACGGTTTGCCAC | AGATGATGTAATCGTTGCACTC |
| Tgfbr2-E4 | GACCTGTTGTTGGTCATTATCC | CGTCCTCCAGGATGATGGC |
| C3 | CAGTTGGGACAACCATAAACC | GAAGTACCTCATGTGGGGCC |
| C4a | CACACCTGGCACCCCCGG | CCGCCTTCGTTTGGAGCCT |
| C4b | GCTGCTGAACTCCATCAGGA | AGACCCGCAACTTCCTGAGC |
| Fcgr2b | CTAAATGTGGTTCTGGTAATCAT | GCCATTGTTATTATCCTAGTATC |
| Fcgr3 | CACTTGTCTTGAGGAGCCTG | CTGTCCAAGACCCAGCAACT |
| LiLrb3 | AGGCTGCCGAATCTGGGGA | CATTGCTCCATGTCCTTGGG |
| Glul (Glutamine synthetase) | GGCTTCTGTCACCGCATAG | GGCGTCTGACTGGATTCCA |
| Casp1 | GTCCCGGGAAGAGGTAGAAAC | GGAGACAT CCTGTCAGGGGC |
| Thy1 | GACTACTTT TGTGAGCTTCAAG | CACAGAGAAATGAAGTCCAGG |
| H2-T23 | GCTTCTGAGGCCAGTCAGA | CCTCCATCCACTGTCTCCA |
| Serping1 | CCTGGGGTCATATACACGAC | CCTGTGCGGGCTGACCG |
| H2-d1 | CACGCTTTACAATCTCTGGAG | CACCCTGAGATGGGAGCCT |
| Ggta1 | CAGACATTATTTCTAACCAAATTAT | GAAGCCCAGTGGCATGATG |
| Gbp2 | CAGAGTATAGTGCACTTCCC | GCATGTGAAGAAGCTGACTG |
| Fbln5 | CAGAACGGATACTGCGACAC | CCAGATCAAATCTGGCAACG |
| Ugt1a1 | CAATGGGTCTTGGATTTGTGT | CGTGATGAGGCACAAGGGG |
| Fkbp5 | CATACATGGCCTTTGGCCTT | CGCCTGCAGATCTCCATGT |
| Psmb8 | CACAGAGCGGCCTCTCGG | CCTGAAGAGGCCTACGACC |
| Srgn | CGTCTTCTGGTTGGTCTTGG | GACTATGGGTCAGGTTCGG |
| Amigo2 | CTAAGTGGATGCCACAAAGG | GCCTCTGCTGACGATCGGA |
| Clcf1 | CAGAAACCATGGGCCTCCAA | GCCCCAGCCTCTGCCAG |
| Tgm1 | GCTCCACCTCGATATGCCAT | CTGCGCCAGACGTTTGTTC |
| Ptx3 | CATACTGGGCTCCTCCGTG | GGCTTTGACGAATCATTAGCAT |
| S100a10 | CTACTTCTTCCCCTTCTGCTT | GGACCCTCTGGCTGTGGAC |
| Sphk1 | GGTTCTTCTGGAGGTGGCC | GGCAAGCATATGGAACTTGAC |
| CD109 | CAATGTTGCACAAAGTACAGAAG | CCAAGGAGACAGGCAGTGC |
| Ptgs2 | CAGCTCAGTTGAACGCCTTT | GGAGGCGAAGTGGGTTTTAAG |
| Emp1 | CTTCCTCAGGACCATATAGAG | CCGGTTCTTCCTCTCAGGG |
| Slc10a6 | CTATTCACATGAAGGAATGTGG | GTCGCAGCATATCAGGCATAC |
| Tm4sf1 | GCAGTTATATTGCTGTTGGCG | CAGTACCTTCTGAATTCCTCTA |
| B3gnt5 | CTAAGCAAATGCAGCCCAAC | GATGACATCTCACGGACACTT |
| CD14 | CAAAGAGGCGATCTCCTAGG | CTCAGCGTGCTGGATCTCAG |
| Fn1-7683 | CTCTCGAGAATCGTCTCTGTC | GGCTGGCGCTGTGACAACTG |
| Slpi-621 | GGCAGGCAGACTTTCCCAC | GAGGTGCTGCCAAGATGCTT |
| Saa3-402 | CAGTATCTTTTAGGCAGGCCA | GCCTGGGCTGCTAAAGTCAT |
| Cfp-1493 | GGGTTTCTTCTCTTCTGGGTC | CCAAGTACCCGCCTACAGTT |
| Pf4-465 | CTAACTCTCCAGGATTTTCTTG | CTGGGATCCATCTTAAGCACA |
| CD5l-1179 | CACACATCAAAGTCTGTGCAG | GAAGTCCCTCCATCCATCC |
